# Teledermatology for Enhancing Skin Cancer Diagnosis and Management: Retrospective Chart Review

**DOI:** 10.2196/45430

**Published:** 2023-05-29

**Authors:** Julia L Gao, Amanda Oakley

**Affiliations:** 1 Department of Medicine Dartmouth Health Lebanon, NH United States; 2 Waikato Clinical Campus University of Auckland Hamilton New Zealand; 3 Department of Dermatology Te Whatu Ora Waikato Hamilton New Zealand

**Keywords:** teledermatology, melanoma, keratinocyte cancer, basal cell carcinoma, BCC, squamous cell carcinoma, SCC, referral and consultation, dermoscopy, telemedicine, skin cancer, diagnosis, management, treatment, skin lesion

## Abstract

**Background:**

Skin cancer rates are at all-time highs, but the shortage of dermatologists compels patients to seek medical advice from general practitioners. A new referral pathway called the Suspected Skin Cancer (SSC) service was established to provide general practitioners in Waikato, New Zealand, with rapid diagnosis and treatment advice for lesions suspicious for skin cancer.

**Objective:**

The aim of this study was to assess the quantity, quality, and characteristics of referrals to the SSC teledermatology service during its first 6 months.

**Methods:**

A retrospective chart review of all referrals sent to the SSC teledermatology service during the first 6 months of its operation was conducted. Time to advice, diagnoses, diagnostic discordance, adherence to advice, and time to treatment were recorded. Diagnostic discordance between general practitioners, dermatologists, and pathologists was calculated.

**Results:**

The SSC service received 340 referrals for 402 lesions. Dermatologists diagnosed 256 (63.7%) of these lesions as benign; 56 (13.9%) were histologically confirmed as malignant, including 19 (4.7%) melanomas. The overall discordance between referrer and dermatologist on specific and broad (ie, benign or malignant) diagnoses for 402 lesions was 47% and 26% (κ=0.58, SD 0.07), respectively; 44% and 26% (κ=0.61, SD 0.15) between referrer and pathologist; and 18% and 12% (κ=0.82, SD 0.12) between dermatologist and pathologist. The mean time between referral submission and receiving advice was 1.02 days. The average time to action (eg, excision) was 64.8 days.

**Conclusions:**

An electronic referral system can be an effective form of teledermatology for providing prompt diagnosis and management advice for benign and malignant skin lesions.

## Introduction

The global incidence of melanoma and keratinocyte skin cancers is increasing, and New Zealand is home to one of the highest rates of skin cancer in the world [1,2]. However, there is a severe shortage of dermatologists to diagnose and manage these conditions [3]. At the time of this study, only 2 full-time equivalent public dermatologists were employed to serve over 400,000 residents living in the Waikato region of New Zealand.

As a result, most patients with skin disorders consult general practitioners (GPs), who provide over 85% of dermatological consultations but possess inferior diagnostic accuracy compared with dermatologists [3,4].

This diagnostic uncertainty of nonspecialists drives unnecessary referrals to dermatologists and the needless excision of benign lesions. Teledermatology, a form of telemedicine consisting of remote consultations with a dermatologist, can mitigate this by providing easier, faster access to a dermatologist’s opinion. It reduces waiting times, produces cost savings, and leads to satisfied patients [5-7]. This is especially important for patients with skin cancer, especially melanoma, because early intervention is linked to reduced costs and better outcomes [8].

New Zealand has offered a publicly funded teledermoscopy service known as the Virtual Lesion Clinic (VLC) since 2010. At the VLC, nurses record a targeted history and take dermoscopy images of skin lesions, which are important for lesion referrals to dermatology [6,8-10]. This high-quality diagnostic service allows for faster management of skin cancers, but its disadvantages include long waiting times for imaging, requiring patient travel to an imaging centre, and the lack of integration with GP and hospital electronic medical records.

In July 2017, the existing web-based electronic referral system was adapted to include an alternative option for GPs, the Suspected Skin Cancer (SSC) pathway, a new teledermatology service created based on data fields from the British Primary Care Commission’s Quality Standards for Teledermatology [11].

Referrers to the SSC pathway are asked to attach regional, close-up, and dermatoscopic images of up to 5 lesions of concern. In addition, optional, free, and web-based or in-person training in lesion photography and dermoscopy was made available. Unlike VLC referrals, SSC referrals and responses are retained in the patients’ medical records.

The referral is viewed by a consultant dermatologist using a high-quality monitor. The standard referral triage form was modified to allow the dermatologist to select a diagnosis from a menu of International Classification of Diseases-10 codes and to provide advice. Treatment recommendations may include no action, excise, perform a diagnostic biopsy, prescribe a specified medication, cryotherapy, refer to the plastic surgical service, rerefer to the Suspected Skin Cancer service after an interval of time or with better images, or refer to the nurse-led teledermoscopy service for expert imaging. The referrer remains responsible for patient care.

## Methods

### Ethical Considerations

The objectives of this pilot study were to assess the quantity, quality, and characteristics of referrals to the Suspected Skin Cancer teledermatology service during its first 6 months. As a service review, it was exempt from requiring approval by a New Zealand Ministry of Health’s Health and Disability Ethics Committee.

### Overview

**Figure 1:**
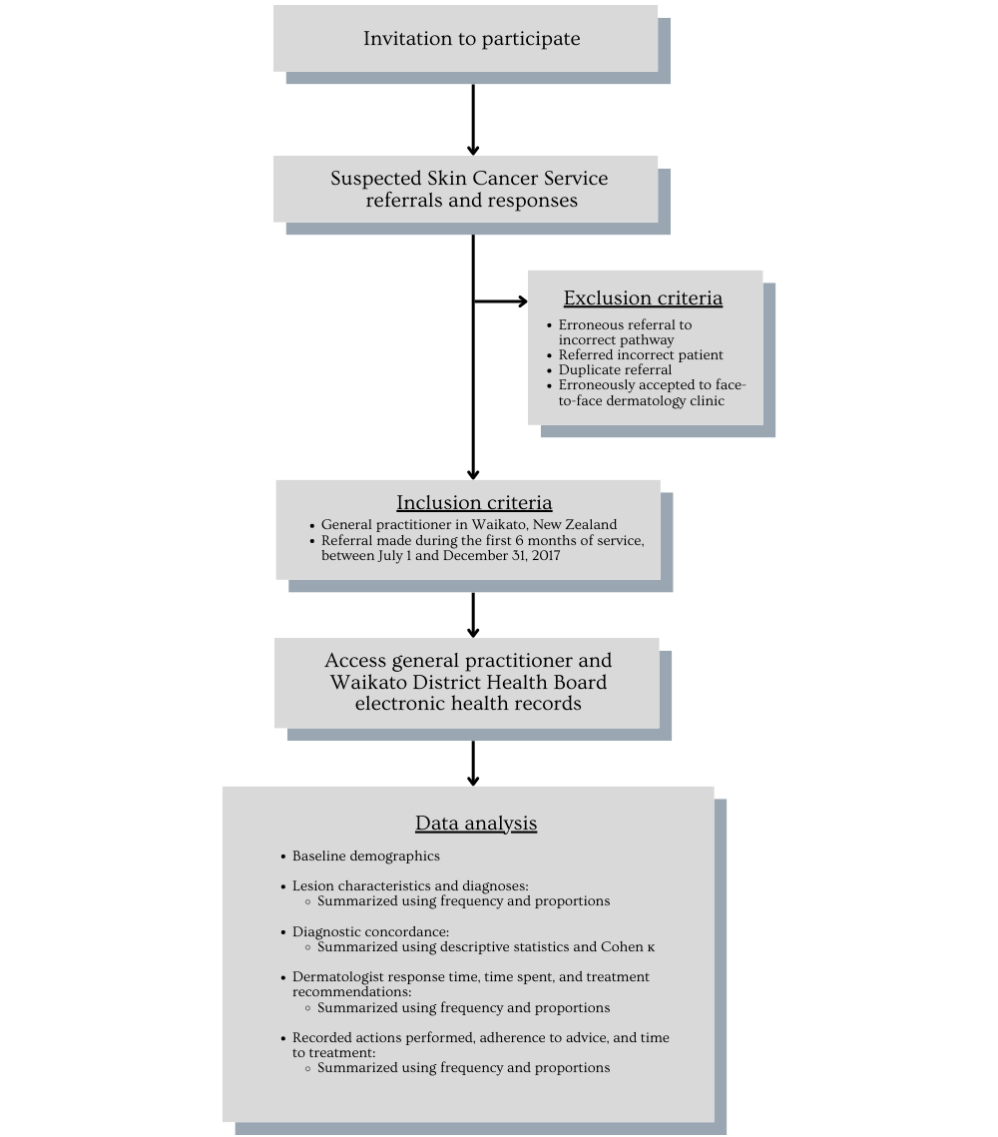
Study flowchart.

We conducted a retrospective chart review of all patients referred to the SSC service between July 1 and December 31, 2017, using electronic health records at Waikato Hospital, formerly known as the Waikato District Health Board, and summarized GP electronic records. Lesion outcomes were followed until July 31, 2018. Exclusion criteria included erroneous referral to the incorrect pathway, referral of the incorrect patient, duplicate referral, or erroneous acceptance to face-to-face dermatology clinic (Figure 1).

Referrals and responses to referrals, dermatology clinic letters, summarized primary care records, and histology reports were used to determine the referrer’s diagnosis for each lesion, the dermatologist’s diagnosis for each lesion, the pathologist’s diagnosis of biopsied or excised lesions, lesion details, the dermatologist’s recommendations, and the dermatologist’s response time. The dermatologist’s time spent responding was determined for a sample of referrals.

Diagnostic concordance was based on the specific diagnosis made and by the broad diagnostic category (ie, benign or malignant). For each lesion, the referrer’s presumptive diagnosis was compared to the dermatologist’s diagnosis. For lesions that were biopsied or excised, referrer and dermatologist diagnoses were compared to the pathologist’s diagnosis, respectively. Nonconcordance indicated that the different physicians disagreed upon the diagnosis. Partial concordance was noted when either the referrer or the dermatologist provided multiple diagnoses, one of which was concordant. Concordance could not be calculated when images were inadequate for diagnosis, when the dermatologist did not give a diagnosis, or when the dermatologist noticed an additional significant lesion in a photograph that the referrer did not deliberately mention in the referral. Concordance based on broad diagnostic category (ie, benign or malignant) was quantified using the Cohen , a measure of interrater agreement that accounts for the possibility of agreement occurring by chance.

For many cases, limited access to summarized GP electronic records was possible, and thus adherence to the dermatologist’s advice (the action) could be estimated. The actions evaluated included the prescription of a specific medication or biopsy of the lesion. The time from the date of referral to the date of the action was calculated.

## Results

### Overview

A total of 350 referrals were received between July 1 and December 31, 2017. Of those, 8 were excluded due to referral error, and 2 were excluded due to pathway allocation error. Outcomes of the referrals were followed until July 31, 2018, and so the observation period was 7-13 months after the referral.

### Referrals

There were 340 referrals for 402 unique lesions in 310 individual patients (Table 1). There was an average of 1.2 lesions per referral (range 1-4 lesions). A total of 26 patients were referred multiple times, with 11 patients being referred for the same lesion and 18 patients being referred for a different one.

When the referrers provided images that were suboptimal in number or quality (42/340, 12.4% of referrals), the dermatologist offered suggestions about how to improve the quality of future referrals.

**Table 1:** Patient characteristics.

Suspected Skin Cancer service characteristics		Values
Referrals, n		340
Patients, n		310
Lesions, n		402
**Gender, n (%)**		
	Male	135 (43.5)
	Female	175 (56.5)
Age (years), median (SD; range)		62.9 (19.6; 0.9-99.5)
**Ethnicity, n (%)**		
	European	290 (93.6)
	Māori, Samoan, or Tongan	24 (7.7)
	Asian	6 (1.9)
	Mixed	9 (2.9)
	Not available	1 (0.3)

### Lesion Location

Lesions were most often located on the face (110/402, 27.4% of all lesions), back (77/402, 19.2%), leg (52/402, 12.9%), or thorax (52/402, 12.9%). Lesions were less often located on the arm (34/402, 8.5% of all lesions), other parts of the head and neck (15/402, 3.7%), foot (15/402, 3.7%), or hand (14/402, 3.5%).

### Time to Advice

The time to advice was calculated as the time from receipt of the referral to the dermatologist’s response. The average and median time to advice was 1.02 days and 0.84 days, respectively, and the range was 0.01-4.90 days.

A random sample revealed that the dermatologist took 6 minutes on average to complete a teledermatology consultation (n=10).

### Diagnostic Concordance

The diagnoses made by the referrer, the dermatologist, and, when applicable, the pathologist, were noted and compared for each lesion (Table 2).

Diagnostic concordances were determined for the specific diagnosis (eg, melanoma and seborrheic keratosis) and for the broad diagnostic category (ie, benign or malignant; Table 3). The overall discordance between the referrer and the dermatologist on specific and broad diagnoses for 402 lesions was 47% and 26% (κ=0.58, SD 0.07), respectively; 44% and 26% (κ=0.61, SD 0.15) between referrer and pathologist; and 18% and 12% (κ=0.82, SD 0.12) between dermatologist and pathologist. Histopathological data were missing for 11 suspected skin cancers that were recommended for excision.

Referrers made the same specific diagnosis as the dermatologist for 150 (37.3%) of the 402 lesions. Of the 103 lesions where referrers and dermatologists disagreed on whether the lesion was benign or malignant, 96 (93%) lesions were diagnosed by the dermatologist as benign, and 6 (6%) lesions were diagnosed by the dermatologist as malignant.

Of the 77 lesions the referrer diagnosed as melanoma, the dermatologist diagnosed 16 (20.8%) as melanoma, 25 (32.5%) as melanocytic nevus, and 14 (18.2%) as seborrheic keratosis.

Of the 82 lesions the referrer diagnosed as basal cell carcinoma, the dermatologist diagnosed 33 (40.2%) as basal cell carcinoma, 14 (17.1%) as squamous cell carcinoma, 6 (7.3%) as actinic keratosis, 5 (6.1%) as seborrheic keratosis, 3 (3.7%) as benign melanocytic nevus, and 8 (9.8%) as other benign lesions.

Of the 62 lesions the referrer diagnosed as squamous cell carcinoma, the dermatologist diagnosed 24 (38.7%) as squamous cell carcinoma, 11 (17.7%) as actinic keratosis, 16 (25.8%) as basal cell carcinoma, and 7 (11.1%) as a different benign lesion.

Diagnostic discordance between the referrer and pathologist was observed in 18 excised lesions, of which 9 (50%) were given a benign diagnosis by the referrer whereas the other 9 (50%) were given a malignant diagnosis by the referrer. Discordance between dermatology and pathology was observed in 8 lesions (Table 3).

**Table 2:** Diagnoses of referred lesions.

Lesions		Referrer diagnosis^a^		Dermatologist diagnosis^a^			Pathologist diagnosis^b^	
		n	Ratio^c^ (%)	n	Ratio^c^ (%)	n		Ratio^c^ (%)
**Malignant**		235	56.0	130	33.7	56		74.7
	Melanoma	77	18.3	25	6.5	19		25.3
	Basal cell carcinoma	82	19.5	59	15.3	26		34.7
	Squamous cell carcinoma	62	14.8	44	11.4	11		14.7
	Merkel cell carcinoma	0	0	1	0.3	0		0
	Not specified	14	3.3	0	0	0		0
**Benign**		185	44.2	256	66.3	19		25.3
	Melanocytic nevus	96	22.9	101	26.2	3		4.0
	Vascular lesion	8	1.9	24	6.2	0		0
	Other inflammatory lesion	6	1.4	17	4.4	3		4.0
	Other nonmelanocytic benign lesion	50	11.9	109	28.2	13		17.3
	Not specific	25	6.0	5	0	0		0
Total		420	100	386	100	75		100

^a^In some cases, multiple diagnoses were made for one lesion. Each diagnosis was counted.

^b^Pathologists only diagnosed lesions that were excised or biopsied.

^c^The ratio is the number of diagnoses of a particular category compared to the total number of diagnoses given.

**Table 3:** Diagnostic concordance.

Diagnosis		GP^a^ vs dermatologist	GP vs pathologist	Dermatologist vs pathologist
**Specific diagnosis, n (%)**				
	Concordant	150 (37.3)	35 (50.7)	46 (69.7)
	Nonconcordant	189 (47.0)	30 (43.5)	12 (18.2)
	Partially concordant	28 (7.0)	4 (5.8)	8 (12.1)
	Unable to calculate	35 (8.7)	0 (0)	3 (4.3)
**Broad diagnosis**				
	Concordant, n (%)	235 (58.5)	46 (66.7)	53 (76.8)
	Nonconcordant, n (%)	103 (25.6)	18 (26.1)	8 (11.6)
	Partially concordant, n (%)	31 (7.7)	5 (7.2)	5 (7.2)
	Unable to calculate, n (%)	33 (8.2)	0 (0)	3 (4.3)
	Cohen κ	0.58	0.61	0.82

^a^GP: general practitioner.

### Adherence to Advice

Primary care records showed that the patient’s GP followed the dermatologist’s treatment recommendations for 74.1% (140/189) of the lesions for which the dermatologist recommended action. The SSC dermatologists recommended excision for 78 lesions, of which 60 (76.9%) were excised and another 11 (13%) were on the waiting list for excision surgery at the end of the study period. An additional 3 (3%) lesions were excised that did not receive a dermatologist’s recommendation for excision. Of the 402 lesions, 63 (15.7%) lesions were excised, 35 (8.7%) lesions were prescribed medication, 11 (2.7%) lesions underwent shave or punch biopsy, and 7 (1.7%) were treated with cryotherapy; moreover, 2 (0.5%) lesions were referred to the plastic surgery department for assessment, and 1 (0.2%) lesion was followed up by the GP, as recommended. Referrals with poor image quality led to a recommendation that the GP refer to the VLC or refer again to the Suspected Skin Cancer pathway with higher-quality photographs (46/340, 13.5% of referrals), and this recommendation was followed for 29 lesions (29/46, 63.0%).

For 4 (4/189, 2%) lesions where actionable advice was given, the advice was partially followed—the GP either prescribed a similar medication to that recommended, prescribed only some of the medications recommended, or took longer to rephotograph the lesion than recommended. In 4 (4/189, 2%) cases, data about prescriptions, excisions, or cryotherapy were missing.

In 41 (41/189, 22%) cases where the teledermatologist provided actionable advice, the limited access to primary care records indicated that the recommended advice may not have been followed. Some referrals to the plastic surgery department did not lead to the recommended excision (4/58, 6.9%). Data for prescriptions were missing in 10 (10/36, 27.7%) cases where the dermatologist recommended treatment with medication. We found data indicating patient noncompliance with treatment recommendations (3/189, 1.6% of lesions where actionable advice was given), lesion regression (2/189, 1.1% of lesions), and patient death (1/310, 0.32% of patients).

### Time to Treatment

The average time from the date of the Suspected Skin Cancer referral to the date the action was performed, if any, was 64.8 days (SD 73.1 days; n=144 lesions acted upon; Table 4). The average time from the Suspected Skin Cancer referral to excision performed by different specialties was 108.3 days for plastics (SD 71.9 days; n=42), 43.9 days for GPs (SD 89.1 days; n=17), 61.1 days for dermatology (SD 48.8 days; n=2), and 75.23 days for oncology (n=1). When lesions were recommended to be treated by medication, the median time from Suspected Skin Cancer referral to being prescribed the medication was 2.4 days.

**Table 4:** Time to action by action performed (in days).

Variable	Time to action (days)			
	Average	Median	Minimum	Maximum
Excision (n=67)	88.6	67.9	2.4	374.5
Prescribe (n=35)	12.6	2.4	0^a^	153.6
Rerefer (n=23)	58.8	32.4	0.4	268.2
Biopsy (n=11)	70.7	25.2	8.2	178.6
Cryotherapy (n=7)	60.9	43.3	1.2	182.4
Follow-up by GP^a^ (n=1)	83.3	83.3	83.3	83.3

^a^GP: general practitioner; some of the recommended prescriptions were already prescribed at a recent GP visit before the dermatologist advice was received.

## Discussion

### Principal Results

The Suspected Skin Cancer pathway is an adaptation of a New Zealand public hospital electronic referral system and is intended to expedite the early diagnosis and treatment of skin cancers by increasing access to dermatology advice. We have shown that this form of store-and-forward teledermatology can provide fast and accurate support to primary care physicians when they have any diagnostic or treatment uncertainty about a skin lesion. All referrals were assessed within 5 working days, and many were assessed within a few hours. Whereas the routine wait time for a face-to-face first specialist assessment at the Waikato Hospital’s dermatology outpatient clinic is 120 days, the average time to advice was only 1.02 days via the SSC service, with clear implications for faster and increased access to dermatology advice.

Referrers were almost 5 times as likely to proffer a diagnosis of melanoma compared with the dermatologists for the same lesion. We suspect the Suspected Skin Cancer pathway significantly reduced the number of unnecessary excisions and needless referrals for benign lesions, consequentially saving patients anxiety, expense, and risk of complications. The majority of lesions suspected to be melanoma by the dermatologists were confirmed histologically, which is consistent with our previous research that demonstrated the diagnostic equivalence of teledermatology to in-person evaluations [8]. Referrers were also 2.5 times more likely to diagnose lesions as basal cell carcinoma or squamous cell carcinoma compared with the dermatologists. As almost half of the specific diagnoses made by the referrer were discordant with the dermatologists’ diagnoses, GPs and their patients would benefit from timely access to teledermatology.

The Suspected Skin Cancer pathway shifts some of the patient care burden to primary care. An electronic referral to a specialist service is normally a quick process, as many fields are automatically populated from the practice management system’s database, and care of the patient is handed over to another service, but the responsibility of care for a teledermatology patient is retained by the GP. Most referrers followed the advice given by the dermatologist.

### Limitations

It should be noted that lesions diagnosed as benign by dermatologists were not followed further to verify stability, and this is a limitation of this study.

Although the Suspected Skin Cancer pathway is a free service, referrers need time and equipment—a dermatoscope, a camera, and a secure data transfer method—for referrals to have the appropriate details. Equipment burden can be lessened by obtaining a dermatoscope that can attach to a smartphone, thus eliminating the need for a separate camera. We estimate that referrers typically spend 10 to 15 minutes to complete the referral form, including capturing and uploading images. The patient may also incur costs for additional visits to their GP clinic for imaging and follow-up.

### Comparison With Prior Work

Our previous research reported that referrers greatly value the educational component of a teledermatology consultation, especially because it is often offered within hours of making the referral [12]. The dermatologist’s work of assessing electronic referrals is time-tabled and offers an important opportunity to train dermatology registrars and medical students in teledermatology, teledermatoscopy, and skin cancer identification and treatment.

By reducing the time to diagnosis and retaining management of some cases in primary care, teledermatology often expedites treatment compared to traditional face-to-face encounters. For example, the average time from Suspected Skin Cancer referral to excision in primary care was less than half of that for lesions excised by the plastic surgery service. Increasing the availability of teledermatology services in conjunction with bolstering GP capacity to provide treatment for skin lesions will promote better care for patients, especially in areas with a lack of dermatologists.

### Conclusions

In conclusion, our adaptation of an electronic referral system to provide a teledermatology service has improved patient access to dermatology and has promoted early identification and treatment of skin cancer. It has the potential to reduce waiting lists for in-person appointments and surgery, to expand access to the accurate diagnosis and appropriate management of skin lesions, and to improve productivity.

## Abbreviations

GP: general practitioner
SSC: Suspected Skin Cancer
VLC: Virtual Lesion Clinic
